# Three-dimensional in situ morphometrics of *Mycobacterium tuberculosis* infection within lesions by optical mesoscopy and novel acid-fast staining

**DOI:** 10.1038/s41598-020-78640-4

**Published:** 2020-12-11

**Authors:** Robert J. Francis, Gillian Robb, Lee McCann, Bhagwati Khatri, James Keeble, Belinda Dagg, Brad Amos, Francisco J. Salguero, Mei Mei Ho, Anwen Bullen, Gail McConnell, Kirsty MacLellan-Gibson

**Affiliations:** 1 Biological Imaging Group, Analytical and Biological Sciences Division, National Institute for Biological Standards and Control (NIBSC), South Mimms, Hertfordshire, EN6 3QG UK; 2 Bacteriology Division, National Institute for Biological Standards and Control (NIBSC), South Mimms, Hertfordshire, EN6 3QG UK; 3 Department of Physics, SUPA, University of Strathclyde, 107 Rottenrow, Glasgow, G4 0NG UK; 4 Public Health England, Manor Farm Road, Porton Down, Wiltshire, SP4 0JG UK; 5 University College London Ear Institute, 332 Grays Inn Rd, Kings Cross, London, WC1X 8EE UK

**Keywords:** Biological techniques, Diseases, Pathogenesis

## Abstract

Tuberculosis (TB) preclinical testing relies on in vivo models including the mouse aerosol challenge model. The only method of determining colony morphometrics of TB infection in a tissue in situ is two-dimensional (2D) histopathology. 2D measurements consider heterogeneity within a single observable section but not above and below, which could contain critical information. Here we describe a novel approach, using optical clearing and a novel staining procedure with confocal microscopy and mesoscopy, for three-dimensional (3D) measurement of TB infection within lesions at sub-cellular resolution over a large field of view. We show TB morphometrics can be determined within lesion pathology, and differences in infection with different strains of *Mycobacterium tuberculosis*. Mesoscopy combined with the novel CUBIC Acid-Fast (CAF) staining procedure enables a quantitative approach to measure TB infection and allows 3D analysis of infection, providing a framework which could be used in the analysis of TB infection in situ*.*

## Introduction

Tuberculosis (TB) caused by the bacterium *Mycobacterium tuberculosis* (*Mtb*) remains the world’s biggest killer by an infectious disease and claims around 1.5 million lives in 2018^1^. Resistance to antibiotics is becoming more prevalent^1^ leading to a pressing need for new TB vaccines and treatments. Many candidate *Mtb* drugs and vaccines are under development, and selection of such candidates is first performed by testing in preclinical animal models.

The current approach for testing efficacy of new TB vaccine candidates in animal models relies on microbiological confirmation by culturing *Mtb* from infected organ homogenates. Although this culture method is reliable as a readout, it fails to show the extent of localised granuloma formation. Host outcome for *Mtb* infection is determined by granuloma-specific heterogeneity; one or few granulomas that fail to contain bacteria may be responsible for allowing bacterial dissemination, worsening pathology and driving active disease^2–5^. Therefore, it is essential to understand the extent of TB infection in target organs using microscopy. In small TB infection models such as mice, rabbits and guinea pigs, *Mtb* bacteria present within organs can be visualised and quantified by histopathology following acid-fast staining. This method has previously only been used to perform two-dimensional (2D) measurements. It has now been recognised that examination of a single section results in data loss due to information lost from above and below the observable section, as demonstrated in other pathologies^6^. For 3D measurements it is possible use histopathology serial sectioning^7^ by microtome, however this process is time consuming, requires skilled technicians, and can introduce artefacts due to distortions during cutting and lost sections. Lesions in mouse models can also be too large to visualise at cellular resolution for conventional microscopes as they can range from tens to hundreds of micrometres in diameter^8^. Therefore, novel 3D approaches and techniques are required for allowing quantitative analysis of TB infected tissues.

Tissue clearing techniques do not require physical sectioning, and have recently allowed investigations into 3D structures, for example analysis of neurons in brains^9^ and cancer pathologies^6^. Many techniques have been published for optically clearing tissues, including organic solvent and aqueous based techniques. Solvent based techniques utilise solvents such as BABB^10^ and 3DISCO^11^ that offer fast clearing but could be harmful to the user, produce hazardous waste^12^ and also cause excessive shrinkage of tissue during dehydration steps^13^. Aqueous, hydrophilic based techniques such as CLARITY^14^, PACT/RIMS^15^ and CUBIC^16^ are safer to use, produce good tissue clearing and retain fluorescence signal^12^. Cronan *et al*^17^ demonstrated the use of the PACT/RIMS tissue clearing technique for visualising *Mtb*. However this technique used genetically modified florescent *Mtb*, which can come as a fitness cost to bacteria^18^. Modification of the *Mtb* is also time consuming and might not be practically suitable for the variety of clinical isolates seen^19^, with different *Mtb* isolates effecting observed pathology in preclinical models of *Mtb* infection^20^.

For ex vivo* Mtb* visualisation in a clinical setting, Auramine O/ Rhodamine B stain is a commonly used fluorescent stain, known as “Truant’s stain”, which binds to mycolic acid and is retained after acid decolourisation. Auramine O/Rhodamine B staining has been used to image *Mtb* within 4 µm paraffin sections for histochemistry^21^. As this stain is fluorescent, 3D microscopy techniques such as Confocal Laser Scanning Microscopy (CLSM)^22,23^ or Light Sheet Fluorescence Microscopy^24^ could be used for imaging the stained *Mtb*. However, imaging from standard CLSM and LSFM must be stitched to form an image of the intact tissue as the field of view is small. Stitching is performed using computational stitching technique, which can cause stitching artefacts and threshold differences across the image montage, resulting in data loss. To address these problems, McConnell *et. al.* developed mesoscopy using the Mesolens^25^, a 4 × magnification lens with 0.47 Numerical Aperture (NA). Mesoscopy is capable of imaging a 6 mm × 6 mm field of view with sub-cellular resolution throughout a 3 mm tissue depth, with no requirement for image stitching^25^. It would be possible then to image whole granulomas within one field of view using the Mesolens without the need for stitching.

The aim of this study is to develop a sample preparation, imaging and analysis pipeline to quantify fluorescence signal and assess any measurable differences between *Mtb* strains in infected mouse lung tissues. Others have shown that it is possible to perform volumetric measurements of pathology using CLSM data^6,26^ and have shown thresholding algorithms of colorimetric Ziehl–Neelsen stained, *Mtb* infected samples^27^, but they have mostly relied on manual thresholding methods that may introduce bias. Semi-automated thresholding methods are now widely available, and by incorporating these we hoped to reduce bias and enable a direct comparison between *Mtb* strains.

## Results

### Optical clearing and imaging of *Mtb* infected lungs

**Figure 1:**
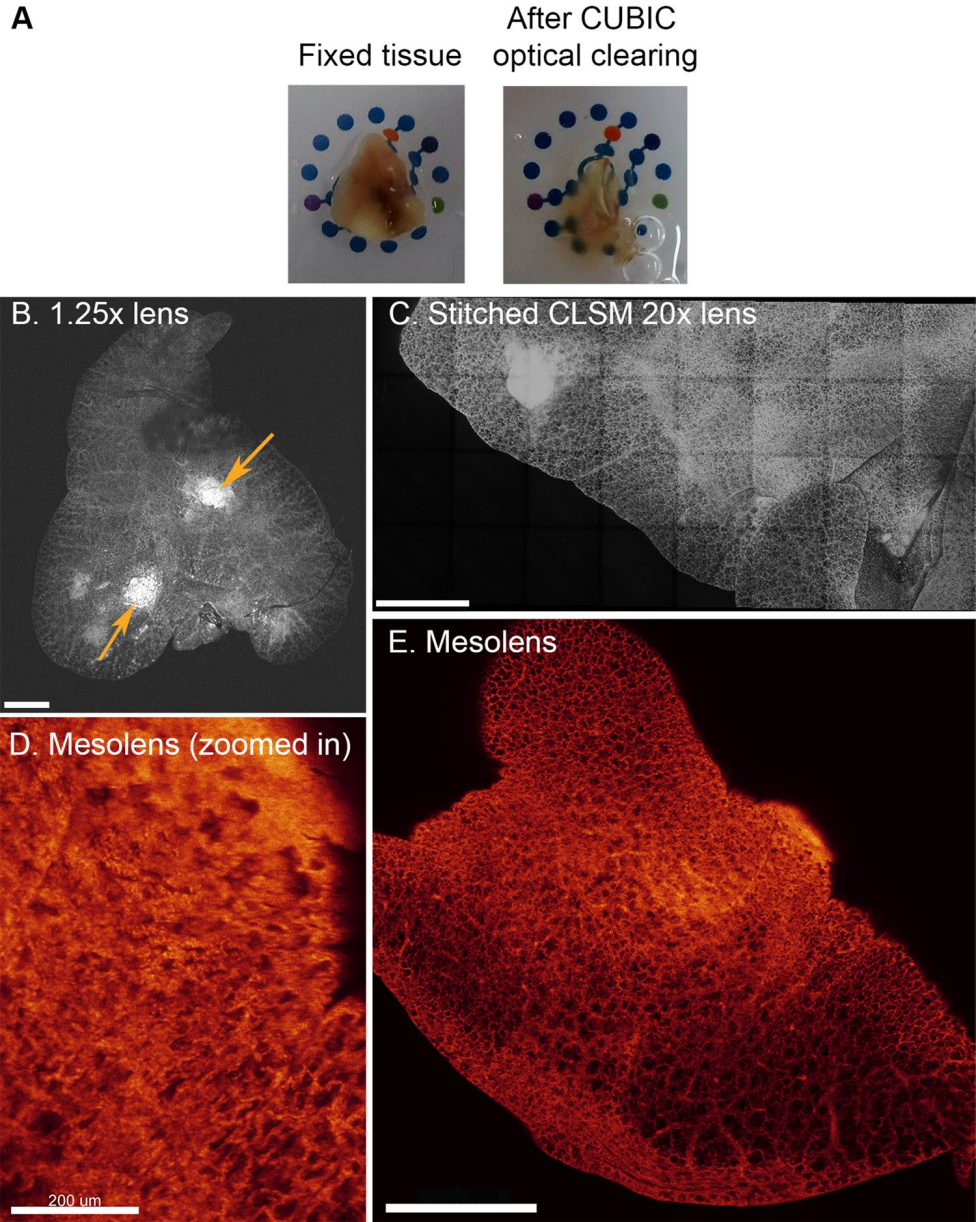
Imaging of optically cleared *Mtb* infected tissue. (**A**) Photographs of *Mtb* infected tissue before and after the scaleCUBIC clearing process. For scale, the NIBSC logo with a diameter of 13 mm is presented behind the cleared lung lobe which was approximately 2 mm thick. Approaches to image optically cleared tissue are presented with imaging by (**B**) a 1.25x /0.04NA lens and (**C**) a 20x /0.75NA glycerol immersion lens. In Mesoscopy (**D**) cellular detail can be seen and (**E**) over a large field of view in 3D without the need to stitch the images. Samples infected with *Mtb* (not specifically stained) and stained with propidium iodide (nucleic acid) for CLSM and Mesoscopy. All images are maximum intensity projections of Z stacked images. Scale bars = 1 mm unless otherwise stated.

To optically clear tissue, the scaleCUBIC^16^ protocol was used. *Mtb* infected lung tissue processed by the scaleCUBIC was shown to be both transparent and intact (Fig. 1A). Imaging by CLSM (using a 1.25X/0.04 NA lens) showed that a piece of tissue stained with a DNA intercalating dye can be imaged, including the visualization of cellular masses presumed to be lesions (Purple arrows, Fig. 1B). Due to the low NA of the lens used, macro structures could be imaged but the lens could not provide any 3D information or image to the resolution required for precise quantification. Imaging using a high NA lens (20x/0.75 NA) resolved cellular detail in 3D, but this required image stitching to image the whole tissue area (Fig. 1C). However, using the Mesolens (4x/0.47NA) cellular detail could be resolved across a 6 mm × 6 mm field of view (Fig. 1D,E) without stitching artefacts. This demonstrated that mesoscopy is a valid technique for imaging optically cleared lung tissue to cellular resolution in 3D.

### Optimisation of Acid-Fast staining of optically cleared *Mtb* infected lungs for confocal laser scanning microscopy

Although imaging nucleic acid stains can be used to visualise gross pathologies (Fig. 1), the Auramine O / Rhodamine B specific stain is required to visualize *Mtb* within the infected tissue. Commonly used stain formulations are optimized for staining sputum samples or thin histological sections^28^ with a high alcohol content (up to 100% v/v). When tested with the cleared samples, this caused extensive shrinkage of fixed tissue after prolonged exposure (data not shown). Phenol content within these formulations is also very toxic for users. To overcome these problems, we developed a new stain formulation, termed CUBIC Acid Fast (CAF) stain, with a high glycerol content and phenylethanol as a phenol replacement (see methods) which minimized damage to the tissue as observed through visual inspection. We also optimised the acid washing of the tissue with an overnight step providing the best removal of dyes apart from that which had bound the acid-fast bacteria (Supplementary Fig. 1), as incubating with acid alcohol for too little time there were no structures visible above the background that had the features of bacterial aggregates. Potassium permanganate is commonly used in Truant’s stain to remove background fluorescence. However, this treatment rendered tissue black and opaque (data not shown) and was incompatible with optical imaging.

**Figure 2:**
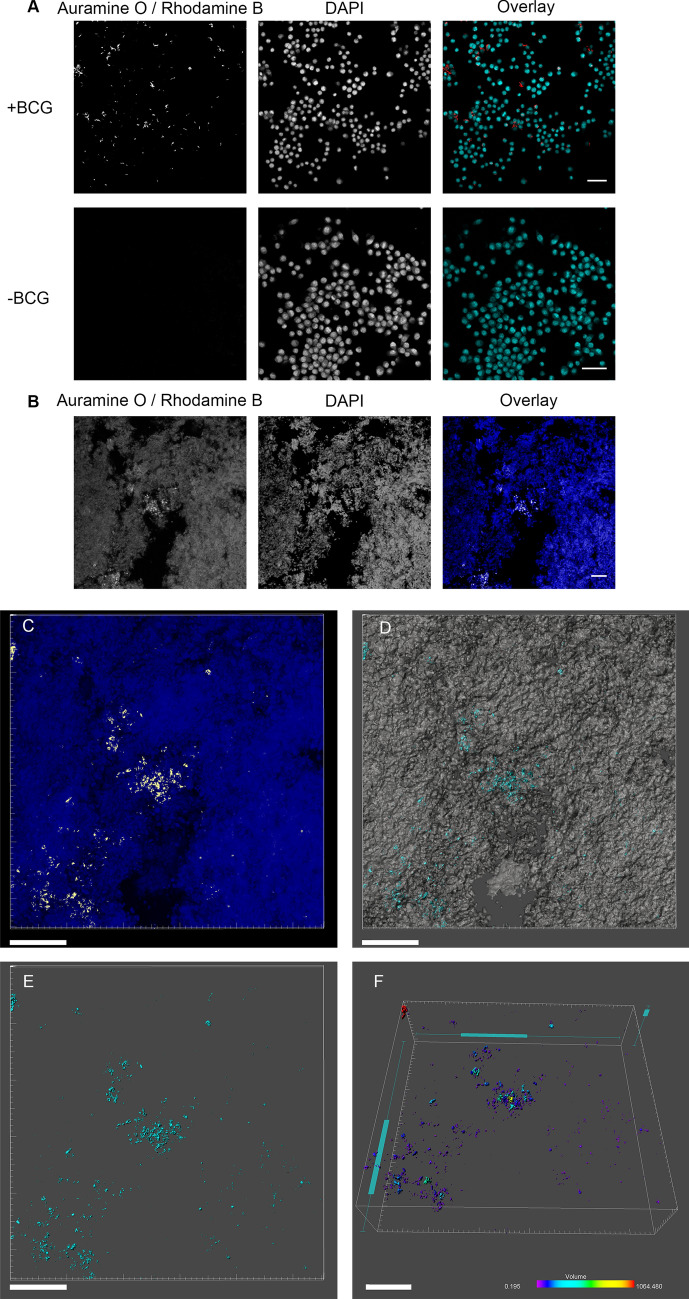
CUBIC Acid Fast (CAF) staining. CUBIC Acid Fast staining procedure demonstrated in (**A**) J774 macrophages infected with or without BCG, and (**B**) in a single CLSM optical section of *Mtb* infected tissue with a non-thresholded image presented. Multiple thresholded optical sections are then constructed into a Z stack (2 µm step size), with a maximum intensity projection presented (**C**) with the AurO/RhodB (yellow) and DAPI (blue) signals visible. (**D**) shows the isosurfaces generated from the AurO/RhodB signal (turquoise) and the DAPI signal (grey), and (**E**) illustrates just the AurO/RhodB signal which the volumes of the bacterial aggregates can be measured, as demonstrated in (**F**) with colony volume colour coded (in µm^3^). Scale bar = 50 µm (**A**, **B**) and 100 µm (**C–F**).

The new formulation was first used on J774 macrophages infected with BCG and counter-stained with DAPI to highlight nucleic acid within the cells (Fig. 2A), with the dye combination used in previous studies^21^. Imaging showed that the CAF stain stained objects that were the expected size of BCG bacilli (approximately 2 µm in length and 0.5 µm in width), as well as bacterial aggregates known to be present within BCG cultures^29^. A control sample containing no BCG showed no specific staining (Fig. 2A).

Having confirmed that the CAF formulation stained the acid-fast bacillus BCG, we then optimised the staining procedure for *Mtb* infected tissue. Slices (500 µm depth) of *Mtb* infected tissue (Supplementary Fig. 2), were optically cleared using the CUBIC protocol, with the CAF specific steps taking place between the scaleCUBIC1 and scaleCUBIC2 incubations (Supplementary Fig. 2). The tissue was then counter-stained with DAPI, placed on a slide with coverslip and imaged using a 20x/0.75NA glycerol immersion lens to closely match the refractive index of the CUBIC2; and with excitation and emission closely resembling the Mesolens parameters. In the optimised preparation, individual bacteria and bacterial aggregates could be visualized in the lung tissue, as seen in the single confocal optical slice (Fig. 2B) and in 3D by confocal Z stack (Fig. 2C). The 3D data from the CLSM can then be used for isosurface rendering (Fig. 2D), with the AuramineO/RhodamineB signal presented (Fig. 2E), and the colony volumes measured (Fig. 2F). CAF staining procedure in conjunction with CLSM was an effective tool to visualise *Mtb* infection within a volume of lung tissue.

### Mesoscopic imaging using Mesolens

**Figure 3:**
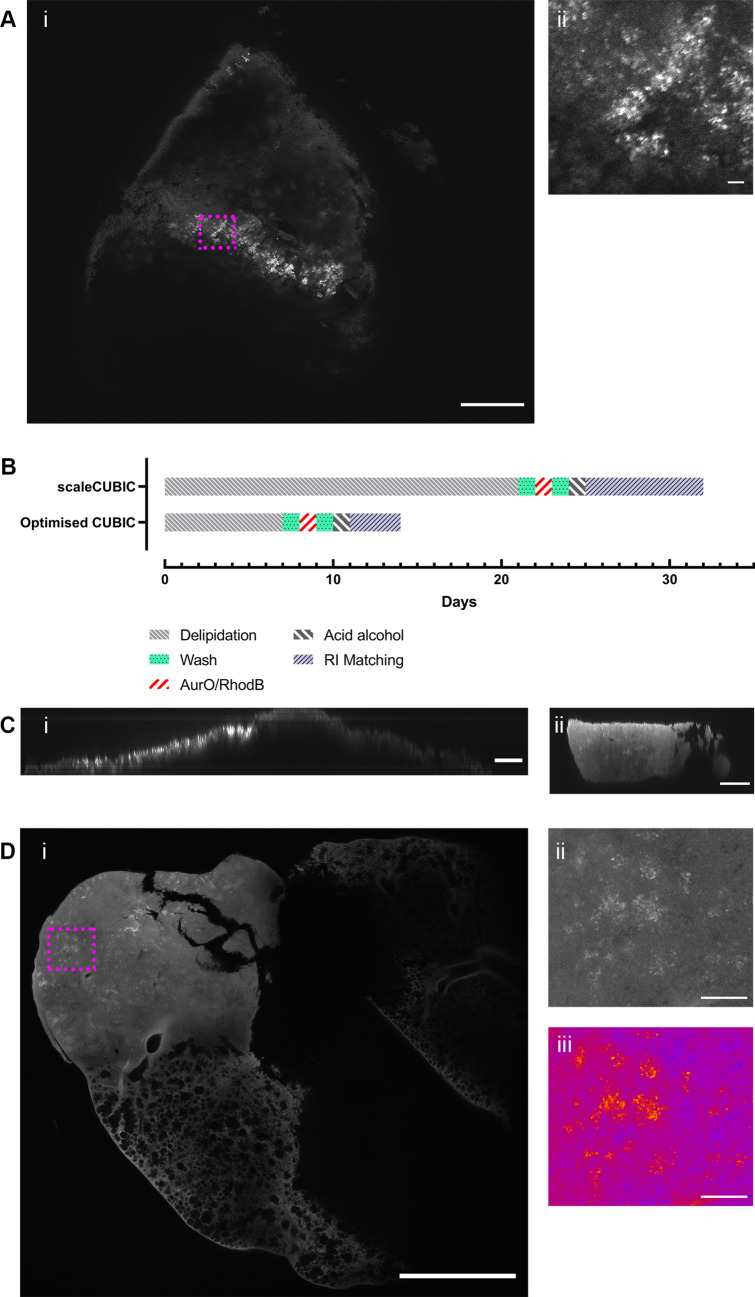
Mesoscopy of *Mtb* infected lung. *Mtb* can be visualized using CAF staining procedure in GC1237 infected mouse lung slice (**Ai**) with a speckly pattern not seen in naïve controls or in tissue without lesions (supplementary Fig. 4) and is present in a zoomed-in image (**Aii**). However, using optimised CUBIC reagents^30^ processing time (**B**) can be reduced and the depth of imaging possible can be increased substantially, with scaleCUBIC (**Ci**) and comparable optimised CUBIC (**Cii**) presented. Using the optimised CAF procedure it is possible to see *Mtb* in a H37Rv infected sample (**Di**) and in zoomed-in images (**Dii**), with pseudo-colouring to highlight the speckled pattern (**Diii**). Scale bar = 500 µm (**Ai**, **Di**), 200 µm (**C**) and 20 µm (**Aii**, **Dii**, **Diii**).

Mesoscopy can be used to microscopically image large pieces of tissue to sub-cellular resolution (Fig. 1). To establish if these images could be used for 3D quantification of *Mtb* in tissues, we used mesoscopy to analyse the optically cleared and CAF stained tissue samples in 3D. Specimens were mounted in the clearing solution for imaging, and the specimens were imaged with oil immersion (Type DF). For imaging simultaneous acquisition was used to visualize the Auramine O and Rhodamine B channels, a 600 nm long pass filter was used for detection as both Rhodamine B and Auramine O have a visible emission above 600 nm. This imaging method did produce background signal, as could be seen in the GC1237 infected lung tissues (Fig. 3A.i), however a “speckly” pattern was visible with granulomatous tissue (Fig. 3A.ii). Individual aggregates could be visualised (Fig. 3A.ii), but resolution was not sufficient to resolve individual bacteria. 3D data was collected (Supplementary Movie 1), and the collected data showed no obvious distortion artefacts from imaging. By comparing the mesoscopy data to routine histopathological methods (Supplementary Fig. 4) it is possible to see the advantages of mesoscopy, with tissue wide analysis at a resolution where individual bacterial aggregates can be seen. This compares to histology where at low magnification the whole tissue can be seen but no bacterial aggregates; or at high magnification stitched images where bacterial aggregates can be seen but with stitching artefacts present (Supplementary Fig. 4). The combination of the mesoscopy and CAF staining procedure revealed the 3D pattern of infection in the tissue from the two strains (GC1237 and H37Rv).

### Further optimisation of the CAF procedure

Although mesoscopy of the CAF stained samples was possible the preparation time was considerable, so further optimisation was required. To optimise the CAF procedure the optimised CUBIC reagents presented by Kubota *et al*^30^ were used. Using CUBIC-L and CUBIC-R reagents the time to process samples was decreased considerably (Fig. 3B), and the depth of imaging was increased. This aided the mesoscopy as a larger depth could be imaged as can be seen with comparable depth (XZ) profiles in scaleCUBIC (Fig. 3Ci) and optimised CUBIC (Fig. 3Cii).

To image whole lesions within tissue mesoscopy was performed on seven prepared infected lung samples, including *Mtb* H37Rv and Kenyan clinical isolates, with 3D data collected as before (Supplementary Movie 2). Interestingly, mesoscopic analysis showed that there were marked differences between mice infected with Kenyan isolates having smaller lesions in comparison to the mice infected with the H37Rv strain (Supplementary Fig. 3). Within lesions, the “speckly” pattern of staining was again present (Fig. 3Di) and individual aggregates could be resolved (Fig. 3Dii, psuedocoloured in Fig. 3Diii). This was not present within non-granuloma tissue or naïve tissue (Supplementary Fig. 5), confirming that *Mtb* aggregates could be specifically visualised within the lesions using the optimised CAF procedure.

### Morphometrics of infection using semi-automated thresholding

Having stained the *Mtb* within lung tissue, we next wanted to see whether bacterial aggregate morphometrics could be measured across intact lesions as previous in vitro studies have shown the significance by linking colony formation to the virulence of pathogen^31^. Morphometrics in this context “deals with the study of form in two- or three-dimensional space”^32^. Analysing morphometrics of *Mtb* aggregates in situ presents a significant challenge as the aggregates are forming in a 3D environment, so 2D methods such as colourimetric histopathology cannot take this into account and may misrepresent the data^21^.

**Figure 4:**
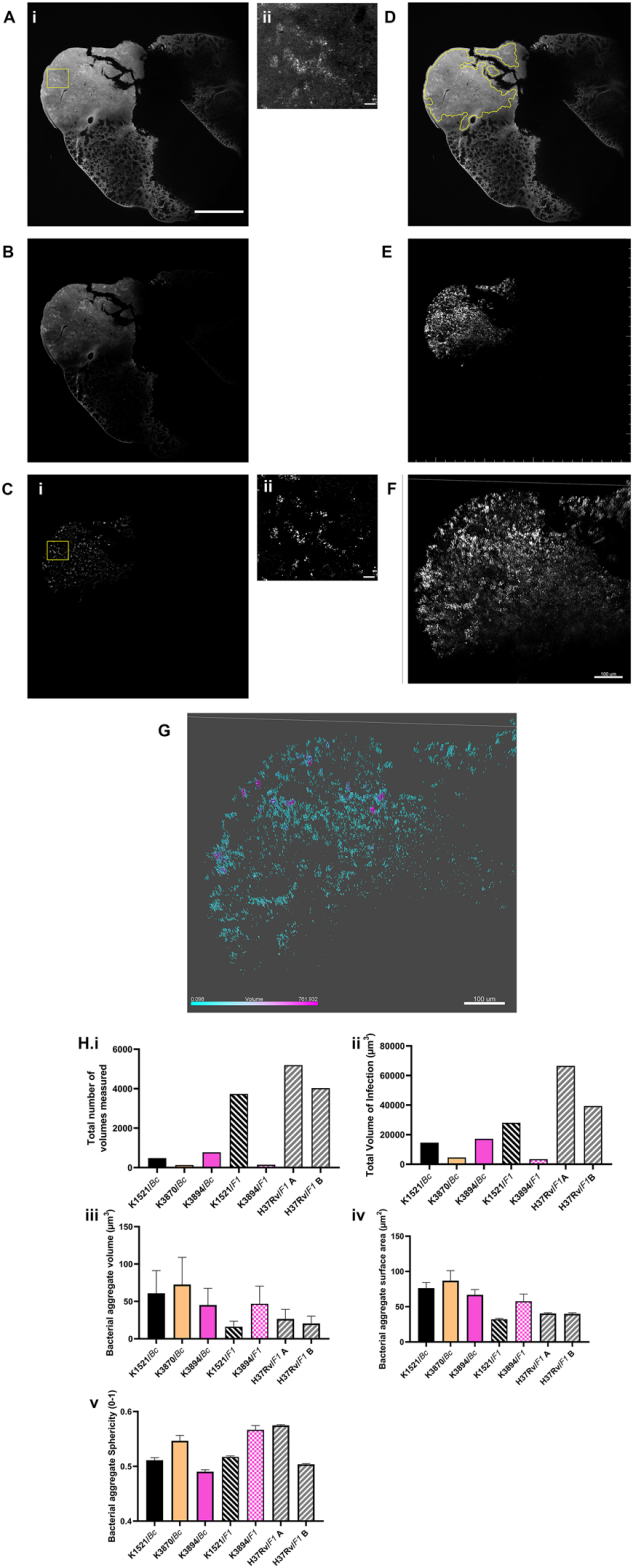
Morphometric analysis of infection within lesions. The steps of the image analysis pipeline are presented (**A–G**). To begin the Mesoscope image (**Ai**) of a H37Rv infected lung sample, with a zoomed-in image (**Aii**) showing the *Mtb* infection as a speckly pattern through the tissue, is mathematically squared (**B**) and the background subtracted (**Ci**). A zoomed-in image (**Cii**) of the yellow region of interest in (**Ci**) shows the *Mtb* pattern that has been isolated. A region of interest is then calculated to cover the lesions only (**D**) and applied to the isolated *Mtb* signal (**C**). The individual Z slices are then constructed into a 3D stack, with a maximum intensity projection of this sample shown in (**E**) and a zoomed-in image at a tilt shown in (**F**). The average RenyiEntropy threshold calculated across Z Stack and Isosurfaces generated (**G**) with the volume (µm^3^) colour coded for visualisation. Examples of morphometric data include total number of volumes (**Hi**), volume of infection within the granuloma ROIs (**Hii**), colony volume (**Hiii**), Surface area (**Hiv**) and Sphericity (**Hv**) in H37Rv and Kenyan isolates infected tissue with volume (individual mice shown with n ≥ 500 volumes measured per stack) (Black = Kenyan 1521, Orange = Kenyan 3870, Pink = Kenyan 3894, Grey = *Mtb* strain H37Rv; Solid colour = BALB/c host strain and Patterned colour = F1 mice). Scale bar = 20 µm (**Aii**, **Cii**) and Scale bar = 500 µm (**Ai**, **B**, **Ci**, **D**, **E**) unless otherwise presented.

To do this, on each optical slice (example Fig. 4A) an image analysis macro (Supplementary Method) was used to form a Region of Interest (ROI) that covered much of the lesions in the tissue (Fig. 4D), as computing limitations prevented the formation of a 3D ROI. A further macro, consistently used through each *Mtb* infected lung, was used to isolate the bright, “speckly” pattern of the *Mtb* (Fig. 4Ci,ii) within the tissue using subtraction of the background signal (Fig. 4B). This created new images of the isolated *Mtb* signal (Fig. 4E) and a RenyiEntropy automated thresholding was applied with the mean threshold generated across the stack (Fig. 4F). The images were then inputted into a 3D image analysis package with isosurfaces calculated^6^ using the mean RenyiEntropy threshold across the stack (Fig. 4G).

Using this method, it was possible to calculate morphometrics of the *Mtb* aggregates within the lesions within individual mice. The H37Rv infection in the two mice showed a larger total number of volumes (aggregates) measured than the mice infected with the Kenyan isolates of *Mtb* (Fig. 4Hi) and a greater total volume of infection (Fig. 4Hii). In contrast the Kenyan isolates seemed to form individual aggregates that were of higher volume (Fig. 4Hiii) and larger surface area (Fig. 4Hiv), while another metric, colony sphericity (Fig. 4Hv), showed minimal differences. Although interesting observations, further data would need to be collected to see if they are biologically significant differences. However, the data collected shows that three-dimensional colony morphometrics can be calculated across tissue imaged by mesoscopy, providing the possibility of further analysis of *Mtb* infection.

## Discussion

Over the past few years optical clearing and 3D fluorescence microscopy has opened the possibility of analysing whole pieces of tissue for pathology, without the need for microtomy^6^. To make the full use of these techniques, tools must be developed to optimally visualize whole tissue pathology. By utilising cheap and readily available small molecule dyes Auramine O and Rhodamine B, this study has demonstrated that *Mtb* infection can be visualized in transparent tissue (by optical clearing technique) and further quantitative analysis can be achieved using 3D fluorescence microscopy and mesoscopy. This work also represents, to the best of our knowledge, the first attempt to provide quantitative 3D measurements of TB infection in situ with maintenance of the spatial distribution of infection.

The choice of tissue clearing technique is an important consideration when attempting to visualize tissue in 3D. There has been a rapid development in this technology over the past decade, with techniques generally falling into two categories; solvent and aqueous based. Solvent based techniques were discounted in this study due to laboratory health and safety reasons, as many include carcinogenic and toxic chemicals for example BABB and DBE^12^, and because solvents can degrade the adhesives within microscope lenses. The aqueous based scaleCUBIC technique^16^ was used, which provided good clearing results without the need for electrophoresis (required by CLARITY). Furthermore, the adaptation of the glycerol wash step with the Auramine O and Rhodamine B dyes enabled incorporation of acid-fast staining within the CUBIC protocol. As this is an original procedure and has been used for the first time to visualise *Mtb* infection in 3D, we have named it “CUBIC Acid-fast (CAF) staining procedure”.

Problems did arise during the development of this procedure. Shrinkage and expansion are common issues of tissue clearing techniques. Direct comparisons between bacterial colony sizes assume that shrinkage and expansion is uniform throughout the tissue. Volumetric measurements in this way could be made with greater accuracy by considering the point spread function of the laser light by 3D deconvolution^33,34^. 3D deconvolution was decided against in this study due to computing limitations but is hoped in the future can be applied to the data generated using CAF staining procedure and mesoscopy.

Although we have demonstrated that CAF staining procedure can be used to visualize and quantify *Mtb* infection in tissues, imaging had to be performed in a specific way by imaging in the single channel with dual excitation at 488 nm and 561 nm and a 600 nm long pass filter. Imaging in this way meant background fluorescence from non-specific binding of the dyes to the tissue and autofluorescence of the paraformaldehyde fixed tissues was present, but minimised. Background non-specific fluorescence is usually removed in Truant’s staining using potassium permanganate solution, but this is not suitable with tissue clearing as the oxidation renders the tissue opaque. Instead, automated image thresholding proved to be an effective solution, enabling a reproducible, non-biased method of quantification of Auramine O and Rhodamine B signals and preferable to manual thresholding. Thresholding assumes that the brightest pixels and voxels within the image were the signal from the bacterial aggregates, which appeared to be the case in comparison to the naïve control samples and CLSM images, and is the same in Formalin Fixed Paraffin Embedded sections^21^. Another contentious issue is that the number of mycobacteria that can be stained by Auramine O and Rhodamine B could impact final volumetric measurements. Recent reports have suggested that only certain populations of *Mtb* strains can be stained with these dyes^35^. Optical clearing procedures that use detergent such as TritonX-100 within the CUBIC-1 and CUBIC-L reagents may also impact on the ability of the Auramine O and Rhodamine B dyes to bind to the mycolic acids that form the cell wall of *Mtb*. Others though have used Auramine O and TritonX-100 successfully^36,37^ albeit with much lower concentrations of TritonX-100. Fluorescence in situ hybridization (FISH) dyes or immunofluorescence could offer a solution for these limitations^35^. However, these techniques would require incorporation into optical clearing protocols.

In this study, we also demonstrated the use of mesoscopy imaging of large pieces of lung tissue. MCLSM is an imaging technique which provides a means of illuminating a single point within the sample, as with confocal laser scanning microscopy, but over a potential 6 mm × 6 mm field of view^25^. Even illumination across a whole plane prevents the need for Z stack stitching, that is required in “Tilescans” used by conventional CLSM, reducing the artefacts present in imaging and analysis. However, the scan speed of confocal laser scanning in mesoscopy is relatively slow, with a frame speed of 3 frames/second. Light sheet fluorescence microscopy^24^ offers a solution by using single whole Z plane illumination rather than the point scanning used in CLSM so increasing the speed of acquisition. However, the field of view is limited as Light sheet microscopy uses lenses that are standard for fluorescence microscopy, so stitching is required for large fields of view^38^ thus introducing the possibility of artefacts and uneven illumination, as with conventional CLSM.

We have shown that 3D mesoscopy and semi-automated thresholding can provide data that is difficult to achieve using other techniques. Histochemical techniques can be used for estimation of infection in 2D but does not consider the heterogeneity on each image plane. While CLSM mitigates this potential error by utilising 3D data stitching is required for large fields of view and the small volume imaged reduces the statistical power. Mesoscopy can determine this across a larger volume, reducing the chances of localisation bias associated with imaging within heterogenous tissue, and without the inclusion of stitching artefacts. Others have shown that colony morphometrics in vitro could provide a way of determining the pathogenicity of the *Mtb* strain^31^. By being able to compare the segmented 3D morphometric data from *Mtb* infected lungs prepared CAF staining procedure could offer a route to study this phenomenon ex vivo.

We predict that the data gathered by the 3D microscopy and mesoscopy could be used to provide additional information from animal models of TB infection, and with techniques and instrumentation such as tissue processors and automated image analysis alongside automated confocal microscopy, the work presented here could provide the framework for high throughput automated analysis of TB infection in vaccine research and drug discovery.

## Methods

### Murine TB aerosol challenge model

Adult female BALB/c or F1 (Hybrid of BALB/c and C57BL/6) mice (10—12 weeks old) were aerosol challenged with low dose *Mtb* strain of H37Rv, Beijing GC1237, or Kenyan clinical isolates 1521, 3870, or 3894, using a Nebulizer System (Walkers, UK) attached with a Middlebrook airborne infection device (Glas-col, Terre Haute, USA). *Mtb* stock was diluted to approximately 5.0 × 10^6^ Colony Forming Units (CFU)/mL for the aerosol generation to achieve an estimated challenge dose of approximately 100 CFU/ lung. Four weeks post-challenge, animals were terminated, and lungs were harvested. Lungs from Naïve mice were also used for comparison. The post caval lobes of the lungs were fixed in 10% (v/v) Formalin (VWR, Lutterworth, UK) overnight. The fixed lung lobes were used for optical clearing and subsequent acid-fast staining with one lobe used for each condition, or for histochemistry and CFU.

### CUBIC Acid Fast (CAF) staining of BCG in J774 cell culture

Adhered, differentiated J774 macrophages were incubated with *Mycobacterium bovis* BCG at a multiplicity of infection of 10:1 for 3 h at 37 °C. The cells and bacteria were washed twice with RPMI (containing 10% Foetal Calf Serum and 1% Glutamine), fixed overnight with 4% formaldehyde and subsequently gently washed with PBS. The PBS was then removed, and the CAF formulation, inspired by the CUBIC procedure^16^ and the Phenol-free Truant’s formulation (Merck Millipore, Burlington, US), containing 50% (v/v) glycerol, 10% (v/v) phenylethanol, 40% (v/v) 2-propanol, 1% (w/v) Auramine O dye and 0.5% (w/v) Rhodamine B (all from Sigma Aldrich, Poole, UK), was added to the cells for 10 min at room temperature. The excess CAF stain was removed by a gentle PBS wash. The acid decolourisation step was performed with 1% (w/v) hydrochloric (HCl) acid in 70% (v/v) 2-propanol in PBS for 1 min at room temperature, followed by washing gently with PBS. The cells were then counterstained with 4′,6-diamidino-2-phenylindole (DAPI, 5 µg/ml) (Sigma Aldrich) in PBS.

### Acid fast (CAF) staining of infected lung tissue

Formalin fixed lung lobes were divided into 0.5 mm slices using a matrix and a razor blade (EMS, Hatfield, PA, USA). The slices were treated with an adapted CUBIC procedure^16^. This involved the incubation of the lung slices in scaleCUBIC1 solution with mixing and daily changes for a week at room temperature. The slices were washed in 50% glycerol in PBS and immersed in the CAF solution for 2 days at room temperature. This was followed by washes with PBS, and immersion in 1% (w/v) hydrochloric (HCl) acid in 70% (v/v) 2-propanol in PBS overnight at room temperature. Afterwards the slices were washed in PBS over the course of a day to remove the acid alcohol. The slices were then immersed in CUBIC2^16^ with regular changes over the course of a week. Then to refractive index match ready for imaging the tissue was immersed in either 80% (v/v) glycerol in PBS, RIMS (88% (w/v) 5-(N-2,3-Dihydroxypropylacetamido)-2,4,6-triiodo-N,N′-bis(2,3-dihydroxypropyl)isophthalamide (Histodenz) (Sigma Aldrich, Poole, UK) in PBS)^15^, fresh scaleCUBIC2 for at least 24 h. The samples were then imaged with the Confocal Laser Scanning Microscopy (CLSM) and Mesoscopy as described below. The same sample was first imaged by CLSM and then imaged by Mesoscopy to confirm the presence of *Mtb*.

### Optimised CAF staining of infected lung tissue

This was performed as above but with the reagents outlined by Kubota et al.^30^. Briefly, 0.5 mm slices were first immersed in CUBIC-L (10% (w/v) polyethylene glycol mono-p-isooctylphenyl ether/Triton X-100 and 10% (w/v) N-buthyldiethanolamine in H_2_O) until clear (around 7 days). The samples were then washed in PBS over the course of a day, and then stained with the CAF reagent for 48 h. The samples were then immersed in 1% (w/v) hydrochloric (HCl) acid in 70% (v/v) 2-propanol in PBS overnight at room temperature. The samples were then washed in PBS over the course of a day, then immersed in CUBIC-R (45% (w/v) 2,3-dimethyl-1-phenyl-5-pyrazolone/antipyrine and 30% (w/v) nicotinamide in H_2_O).

### Confocal laser scanning microscopy

CLSM was performed using a Leica SP8 X CLSM MP OPO TCSPC system (Leica Microsystems, Germany). Slices were placed in a dish with a hole made of Agarose and filled with either CUBIC2 or 80% (v/v) glycerol in PBS. Whole pieces of tissue were imaged using a 1.25x / 0.04NA lens, while slices were covered and imaged with a 20x / 0.75NA glycerol immersion lens, using 80% (v/v) glycerol in PBS as immersion media (between coverslip and lens). To image the Auramine O the excitation of 488 nm and emission between 515 and 545 nm were used. To image the Rhodamine B signal, an excitation of 561 nm and emission between 600 and 700 nm were used. DAPI was imaged using 405 nm excitation and emission between 430 and 470 nm.

### Mesoscopy using mesolens confocal laser scanning microscopy

The Mesoscope is a custom-built system to the specifications of McConnell *et al*^25^. To image the specimens, PBS with 80% (v/v) glycerol or CUBIC-R was used within a custom-built holder^39^, and with the specimen placed under a coverslip, and oil immersion (Type DF) used between the coverslip and the lens. Images were obtained simultaneously using excitation wavelengths 488 nm and 561 nm, and emission wavelengths with 600 nm long pass, with a frame average of 3. The presented Z stacks have a 250 nm × 250 nm × 4 µm voxel size with a bit depth of 8 (compressed from 16-bit for image processing using FIJI (ImageJv2, NIH, USA). All samples were imaged with the same excitation and emission parameters for comparable measurement.

### Volumetric measurements

Figure 4 shows the steps that were taken to isolate the “speckly” pattern within the images which was shown to be specific for *Mtb*. In brief, a Region of Interest (ROI) was added to each 2D optical section in the 3D stacks using a FIJI (ImageJv2) macro that covered specifically granulomatous tissue. To reduce computing requirements only optical sections that contained granulomatous tissue were analysed. Then using background subtraction within the macro, the speckly pattern of the TB was isolated within the images, producing 2D images that could then be recombined into a Z stack. A RenyiEntropy^40^ auto-thresholding method using the “Autothresholding” function in FIJI (ImageJv2)^41^ was then used to generate a mean threshold of the brightest voxels across the Z stack. This was interpreted as the *Mtb* specific Auramine O /Rhodamine B signal. The threshold was then used to generate 3D volumes from the rendered isosurface (Imaris 9.1, Bitplane, Switzerland), using a surface detail of 0.1 µm. This same process was conducted on samples infected with each *Mtb* isolate to allow direct, reproducible and non-biased comparisons.

Colony size measurement was performed with the assumption for this analysis that an aggregate of bacilli was derived from a single infectious bacterium. All volumes crossing the edge of the field of view were not included within the analysis.

### Histochemistry and CFU’s

To confirm that the *Mtb* strains and isolates were infectious in the mouse models, histology and CFU’s of lung were performed to confirm the presence of *Mtb* in the lung. The histology was done using the Ziehl–Neelsen colourimetric staining, as described^42^. Images presented were taken using an EVOS XL Core (Thermo Scientific, Waltham, MA, USA) microscope with colour camera. Stitched images at 40 × magnification were produced using the “Stitching” plugin in ImageJv2^43^. CFU’s were calculated from homogenised samples which were serially diluted, plated in duplicate onto OADC supplemented 7H11 agar plates, incubated at 37 °C, and enumerated after 3 to 4 weeks.

### Ethics statement

All animal procedures were approved by the NIBSC Ethics Committee and were in accordance with the UK Home Office (Scientific Procedures) Act 1986 and the Animal Welfare Act 2006. Animals were monitored by trained animal technicians at least once a day, and this frequency could increase if any adverse reactions were observed.

## Supplementary Information

Supplementary Information 1.

Supplementary Figure 1.

Supplementary Figure 2.

Supplementary Figure 3.

Supplementary Figure 4.

Supplementary Figure 5.

Supplementary information 7.

Supplementary Information 8.

Supplementary Information 9.

## Data Availability

The datasets generated during and/or analysed during the current study are available from the corresponding author on reasonable request.
