# Supplementary material for: Three-dimensional in situ morphometrics of Mycobacterium tuberculosis infection within lesions by optical mesoscopy and novel acid-fast staining
Source: Sci Rep. 2020 Dec 11;10:21774. doi: 10.1038/s41598-020-78640-4 (PMC7733456; doi:10.1038/s41598-020-78640-4)
Supplement: Supplementary file 1 — Supplementary Information 1. [file 41598_2020_78640_MOESM1_ESM.docx]

**Supplementary Information Title:**

Supplementary Information: Three-dimensional *in situ* morphometrics of *Mycobacterium tuberculosis* infection within lesions by optical mesoscopy and novel acid-fast staining

**Authors:**

Robert J. Francis^1#^, Gillian Robb^3^, Lee McCann^3^, Bhagwati Khatri^2^, James Keeble^2^, Belinda Dagg^2^, Brad Amos^3^, Francisco J. Salguero^4^, Mei Mei Ho^2^, Anwen Bullen^1,5^, Gail McConnell^3^, Kirsty MacLellan-Gibson^1^

# Supplementary figure legends

**Supplementary Figure 1. Optimisation of the acid alcohol step of the CAF staining procedure.** Maximum intensity projections of *Mtb* infected tissue that had been stained with the Auramine O/Rhodamine B dye step of CUBIC Acid-Fast and subject to (**A**) 30 minutes and (**B**) overnight acid alcohol. (**ii**) and (**iv**) are psuedocoloured images of (**i**) and (**iii**) respectively. Scale bar = 100 µm (**A.i** and **ii; B.i** and **ii**) and 20 µm (**A.iii** and **iv**, **B.iii** and **iv**).

**Supplementary Figure 2. CAF staining procedure.** Photographs of each stage of the CAF staining procedure on *Mtb* infected lung tissue (H37Rv strain shown). For scale, the institute logo with a diameter of 13mm is presented behind the tissue. Each tissue slice is 500 µm in thickness.

**Supplementary Figure 3. Example of the variation of infection across a CLSM Z stack.** Three random Z slices were chosen across a CLSM Z stack of *Mtb* infected tissue (GC1237 isolate shown) and the percentage infection was measured. *Mtb* is in **Yellow** and the DAPI stained tissue in **Blue**. Scale = 50 µm.

**Supplementary Figure 4. Microscopy of histological stained section of *Mtb* infected tissue.** Micrographs of infected tissue stained using the Ziehl-Neelsen procedure. (**A.i**) shows a micrograph taken with a 4x / 0.13NA lens. With a zoomed in image of the **yellow** region (**ii**) showing how the resolution of the lens is not adequate to see the *Mtb* infection. (**B.i**) shows a micrograph where a series of images taken with a 40x / 0.6NA lens have been stitched together to see the infection across a large part of the tissue. At this magnification individual *Mtb* aggregates can be seen in the **red** region (**ii**). However, when stitching images together artefacts can be present such as the mismatch seen in the contrast changed image of the **yellow** region (**iii**). Scale bar = 500 µm (**A.i**), 200 µm (**A.ii**), 100 µm (**B.i.**) and 20 µm (**B.ii** and **B.ii**).

**Supplementary Figure 5. Mesoscopic imaging of *Mtb* infected and naïve mouse lungs.** Maximum intensity projections of the Z stacks produced from the Mesoscopy of the CAF stained lung tissue. (**A**) shows Naïve lung tissue with no tissue that can be seen to be considered to have lesions. (**B**) shows lung infected with Kenyan isolates 1521 (**i**), 3870 (**ii**) and 3894 (**iii**) in BALB/c and F1 mice. (**C**) shows lung infected with H37Rv strain of *Mtb* in 3 different mice. **Orange arrows** highlight tissue that is considered a lesion with the “speckled” *Mtb* signal within (that is not present in naïve (**A**) and non-lesion tissue (**C.iii**)). (**D**) shows *Mtb* infected tissue that was not stained. Scale bar = 500 µm.

**Supplementary Movie 1. Mesoscopy of *Mtb* GC1237 infected mouse lung prepared using CAF.** A fully rotatable Z stack was obtained with the Mesolens with the thresholded *Mtb* data shown as (**Hot**) and the background staining shown in (**Grey**).

**Supplementary Movie 2. Mesoscopy of *Mtb* H37Rv infected mouse lung prepared using optimised CAF.** A fully rotatable Z stack was obtained with the Mesolens with the thresholded *Mtb* data shown as (**Pink**) and the background staining shown in (**Grey**). Isosurface generated using Imaris shown are **colour coded** for volume of each *Mtb* colony. The isosurface volumes were then used in volumetric measurements.

**Supplementary Method. Mesolens Data Analysis Macro.** Presented as .txt file to be run as an IJ1 Macro.
