# Supplementary figures and images for: Three-dimensional in situ morphometrics of Mycobacterium tuberculosis infection within lesions by optical mesoscopy and novel acid-fast staining
Source: Sci Rep. 2020 Dec 11;10:21774. doi: 10.1038/s41598-020-78640-4 (PMC7733456; doi:10.1038/s41598-020-78640-4)

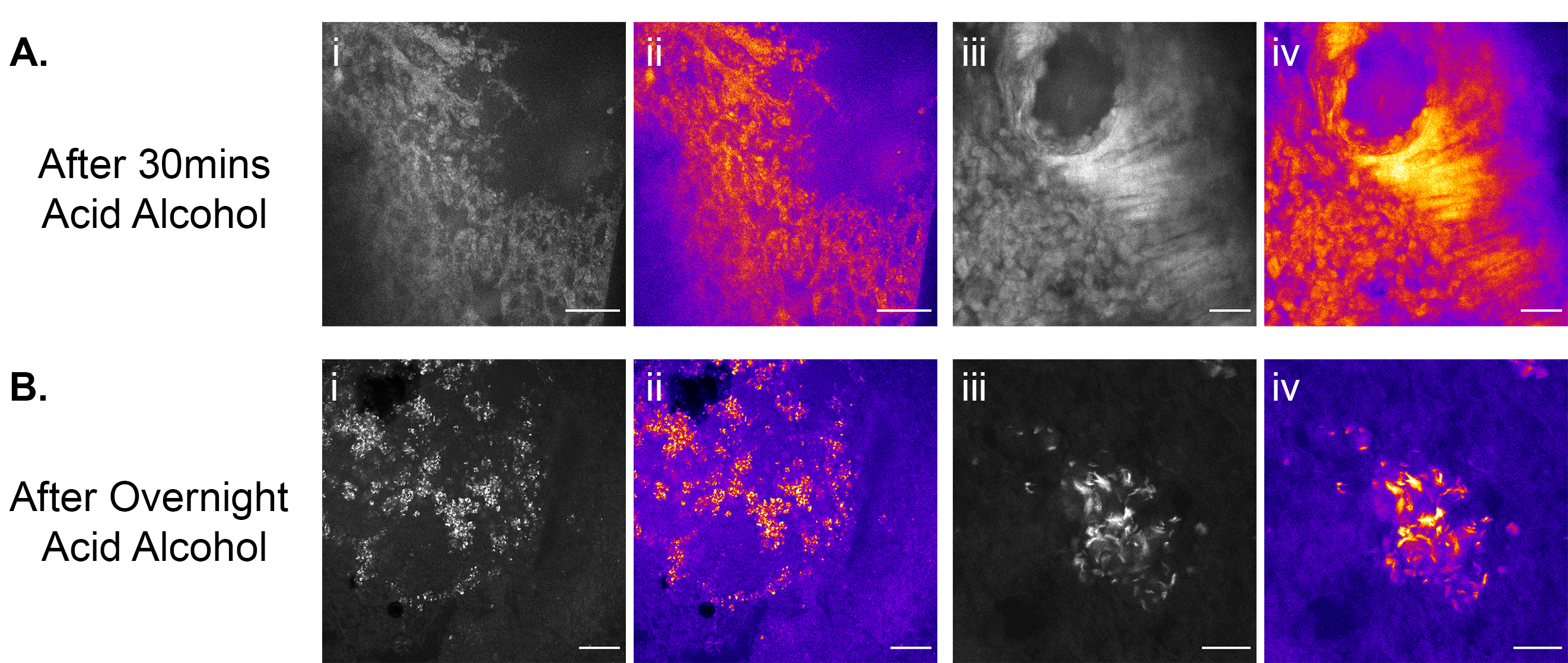

Supplement: Supplementary file 2 — Supplementary Figure 1. [file 41598_2020_78640_MOESM2_ESM.tif]

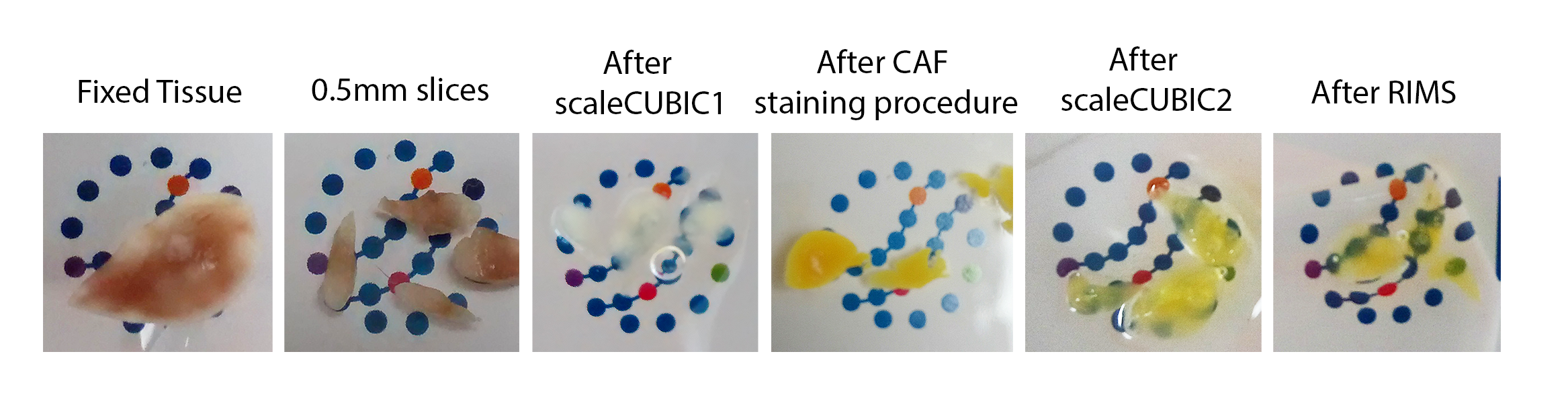

Supplement: Supplementary file 3 — Supplementary Figure 2. [file 41598_2020_78640_MOESM3_ESM.tif]

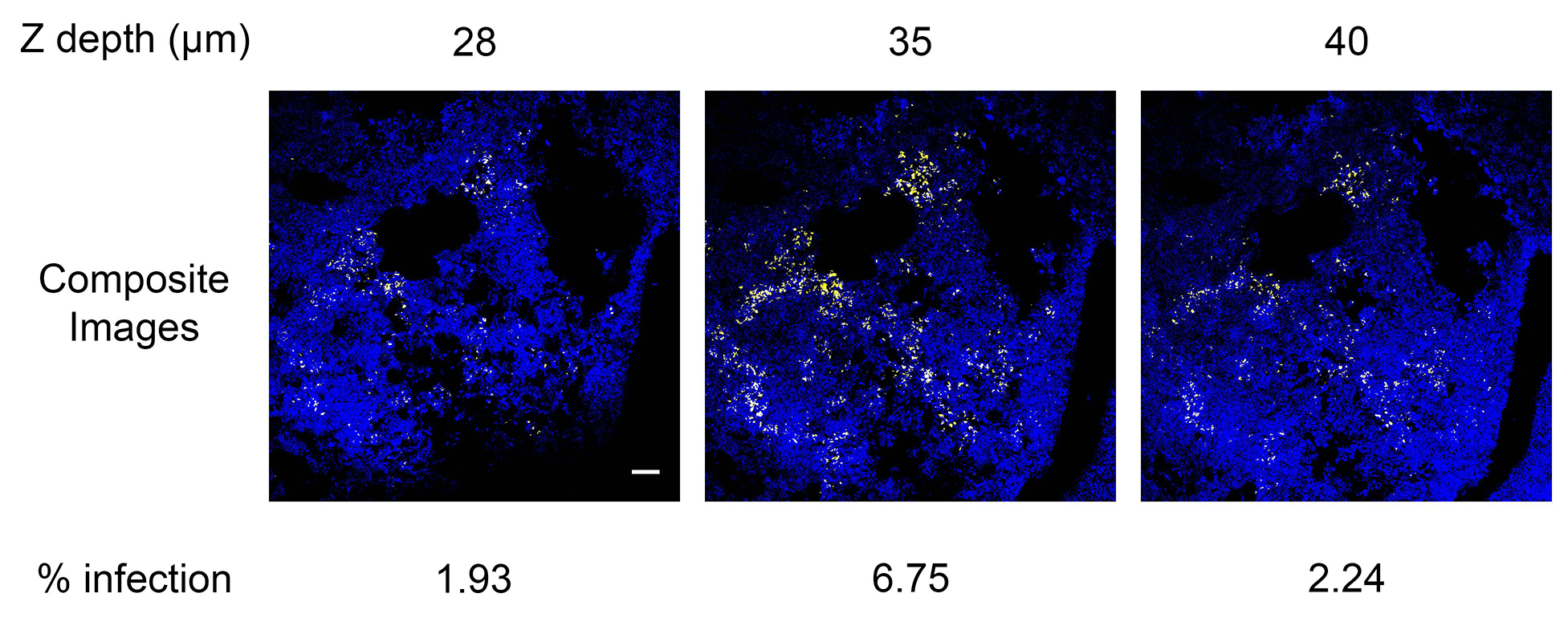

Supplement: Supplementary file 4 — Supplementary Figure 3. [file 41598_2020_78640_MOESM4_ESM.tif]

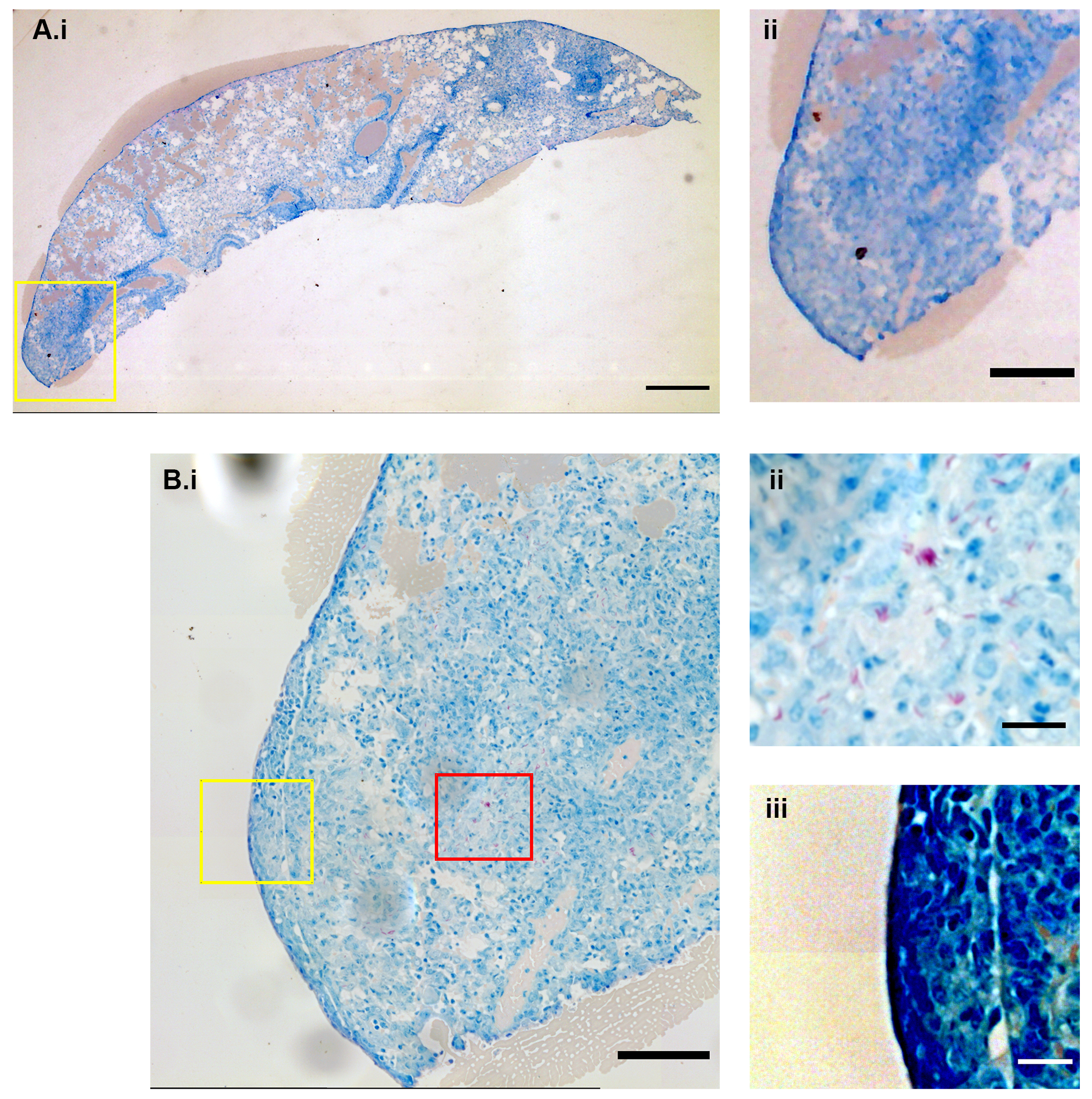

Supplement: Supplementary file 5 — Supplementary Figure 4. [file 41598_2020_78640_MOESM5_ESM.tif]

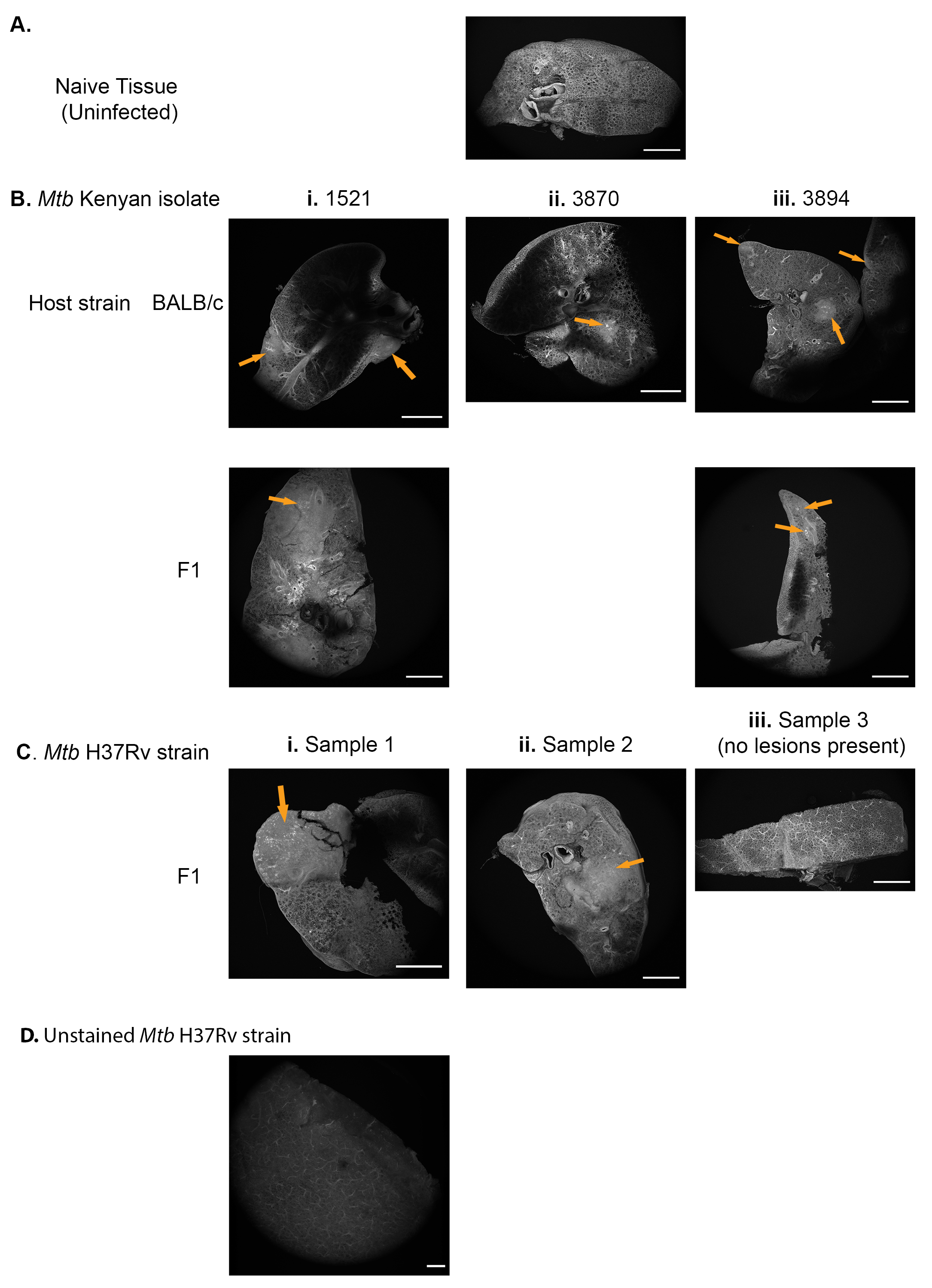

Supplement: Supplementary file 6 — Supplementary Figure 5. [file 41598_2020_78640_MOESM6_ESM.tif]
